# Factors associated with emergency-onset diagnosis, time to treatment and type of treatment in colorectal cancer patients in Norway

**DOI:** 10.1186/s12885-021-08415-1

**Published:** 2021-06-30

**Authors:** Yngvar Nilssen, Morten Tandberg Eriksen, Marianne G. Guren, Bjørn Møller

**Affiliations:** 1 Department of Registration, Cancer Registry of Norway, Postboks 5313 Majorstuen, 0304 Oslo, Norway; 2 Division of Surgery, Inflammatory Diseases and Transplantation, Oslo University Hospital, Oslo, Norway; 3 Institute of Clinical Medicine, University of Oslo, Oslo, Norway; 4 Department of Oncology, Oslo University Hospital, Oslo, Norway

**Keywords:** Colorectal cancer, Emergency presentation, Waiting time, Treatment

## Abstract

**Background:**

International differences in survival among colorectal cancer (CRC) patients may partly be explained by differences in emergency presentations (EP), waiting times and access to treatment.

**Methods:**

CRC patients registered in 2015–2016 at the Cancer Registry of Norway were linked with the Norwegian Patient Registry and Statistics Norway. Multivariable logistic regressions analysed the odds of an EP and access to surgery, radiotherapy and systemic anticancer treatment (SACT). Multivariable quantile regression analysed time from diagnosis to treatment.

**Results:**

Of 8216 CRC patients 29.2% had an EP before diagnosis, of which 81.4% were admitted to hospital with a malignancy-related condition. Higher age, more advanced stage, more comorbidities and colon cancer were associated with increased odds of an EP (*p* < 0.001). One-year mortality was 87% higher among EP patients (HR=1.87, 95%CI:1.75–2.02). Being married or high income was associated with 30% reduced odds of an EP (*p* < 0.001). Older age was significantly associated with increased waiting time to treatment (*p* < 0.001). Region of residence was significantly associated with waiting time and access to treatment (p < 0.001). Male (OR = 1.30, 95%CI:1.03,1.64) or married (OR = 1.39, 95%CI:1.09,1.77) colon cancer patients had an increased odds of SACT. High income rectal cancer patients had an increased odds (OR = 1.48, 95%CI:1.03,2.13) of surgery.

**Conclusion:**

Patients who were older, with advanced disease or more comorbidities were more likely to have an emergency-onset diagnosis and less likely to receive treatment. Income was not associated with waiting time or access to treatment among CRC patients, but was associated with the likelihood of surgery among rectal cancer patients.

**Supplementary Information:**

The online version contains supplementary material available at 10.1186/s12885-021-08415-1.

## Background

Colorectal cancer is the third most common cancer with over 1.8 million new cases worldwide, which represented 10.2% of all new cancers diagnosed in 2018 [1]. In Norway, 4295 patients were diagnosed with colorectal cancer in 2019, representing 12.3% of all cancers diagnosed [2]. Internationally, age-standardised incidence rates vary between countries and ranged from 56.5 in the UK to 81.1 in Denmark per 100,000 [3]. Colorectal cancer represents 9.2% of all cancer-related deaths and is the second most common cause of death in the world [1]. In 2018, 1556 colorectal cancer deaths were registered in Norway, which represents 14.1% of all cancer-related deaths [2]. Net survival has improved over the past few decades [3, 4]. In Norway in 2015–2019, the five-year net survival among colon cancer patients was estimated to be 68.1% in males and 71.1% in females while for rectal cancer it was 71.1 and 71.5%, respectively [2]. Considerable variation in survival has been reported for colon and rectal cancers both internationally and nationally [3–6]. The proportion of patients diagnosed through an emergency presentation (EP), waiting times from diagnosis to treatment, and access to treatment are all contributing factors that may explain some of the observed variation and potentially affect survival.

There are different explanations why patients are diagnosed with cancer following an emergency visit. Some patients may initially have none or vague symptoms, but then suddenly experience a rapid development in symptoms. For others EPs may be a reflection of delayed help-seeking behaviour by the patient (patients delay) or prolonged diagnostic intervals by the general practitioner or the hospital (doctors delay). Internationally, variations exist in the proportion of colon cancer patients (11–39%) and rectal cancer patients (12–16%) who are diagnosed following an EP [7].

The timeliness of and access to treatment are of great importance to a patient’s prognosis. To ensure a timely treatment, sufficient capacity of medical staff and equipment are required, as well as a well-organised and structured health system with clearly defined time frames. In 2015, Norway implemented cancer patient pathways (CPP) in order to 1) reduce unwanted variation in waiting time to treatment, 2) remove non-medically justifiable delays in examination, diagnostics and treatment, and 3) increase the predictability for patients and their relatives [8]. The CPPs consist of recommended diagnostic procedures and a recommendation of the maximum days (for 70% of the patients) that patients should wait from a hospital referral to the first specialist visit (9 days), to a clinical decision (12 days) and finally to the start of treatment (14 days to surgery, 18 days to radiotherapy, 14 days to systemic anticancer treatment (SACT)). With the implementation of CPPs, cancer pathway coordinators were introduced to coordinate referrals, plan appointments, and provide information to the patients until the first specialist consultation. In Norway, it has been shown that over 80% of all colorectal cancer patients were included in a CPP during 2015–2016 [9]. A recent study from Norway showed that the waiting time from diagnosis to surgery for colorectal cancer patients diagnosed from 2007 to 2016 remained around 21 days, while time to start of radiotherapy has decreased by two weeks to 34 days [10]. Studies have also shown that patients with high socioeconomic status (SES) undergo more extensive examinations than low SES patients [11, 12]. This indicates that non-medical factors may affect the waiting time to treatment. Other studies have shown that low SES was associated with a reduced odds of receiving surgery, radiotherapy and SACT both among colon and rectal cancer patients [13, 14].

Hence, the aims of this paper were to describe the pattern of care among colorectal cancer patients in Norway and identify factors associated with EP, waiting time to treatment and access to treatment.

## Methods

### Cancer registry of Norway

Since 1953, it has been mandatory for all hospitals, pathology laboratories and general practitioners in Norway to report all newly diagnosed malignant disease to the Cancer Registry of Norway (CRN). The CRN also receives death certificates for all patients with a cancer diagnosis from the Cause of Death Registry. Using the personal identification number assigned to all Norwegian citizens since 1964, the CRN is linked monthly with the National Population Register to update vital status (death or emigration), and three times per year with the Norwegian Patient Registry (NPR) to ensure completeness of cancer cases. The quality, comparability, completeness, validity, and timeliness of the data in the CRN have been evaluated to be high, with an estimated completeness of 98.8% for all cancer sites together [15].

### Norwegian patient registry

The NPR is a national health register that holds data on all patient visits to government-funded hospitals in Norway. Reporting to the NPR is mandatory, and its database covers over 99% of all patient visits to specialised health care services [16]. These also include data regarding CPPs. From 2008 the NPR data also include personal identification numbers, thus enabling researchers and health authorities to follow the disease trajectory of patients between different sectors and hospitals.

### Statistics Norway

The national statistics institute, Statistics Norway, holds individual-level information in areas such as population, health, finance and education for the entire Norwegian population. Education data have been collected from various national databases since 1970. The tax authorities provide Statistics Norway with personal income data, which are available from 1967, and household type and income, which are available from 2004 onwards.

### Data linkage

The study population included all patients with a colorectal (ICD-10 code C18–20) cancer diagnosis registered at the CRN between 1 January 2015 and 31 December 2016. As described elsewhere, information from the NPR was linked to identify which patients were included in a CPP, the patient’s level of co-existing diseases (i.e., comorbidities) and all registered episodes from the specialist health care [9]. Information about the patient’s SES, measured through household income and education, was obtained from Statistics Norway.

### Classification of variables

As described elsewhere, date of diagnosis was defined as the date of the first histologically verified diagnosis registered at the CRN, which most often was based on a biopsy [9]. For patients whose tumour was not morphologically verified, the date of diagnosis was set as the date from the clinical notification form.

### Stage

Stage of disease was categorised as localised, regional, metastatic, or unknown [17]. For staging, notifications received within the diagnosis period at the CRN, defined as the month of diagnosis plus an additional four months, were used.

### Region

As described elsewhere, Norway consists of four regional health authorities that are responsible for specialised health care in their catchment areas: Southern and Eastern Norway, Western Norway, Central Norway and Northern Norway [9]. Regional affiliation was based on a patient’s place of residence at the time of diagnosis, independent of where the patient was diagnosed or treated.

### Socioeconomic (income, education) and marital status

A patient’s SES was measured using individual information about household income and the highest level of obtained education. Household income included wages, self-employment capital income, pension, and social benefits earned the year prior to diagnosis. The equivalised household income (square root scale), a measure adjusting for the number of people living in the household, was used and grouped as low, intermediate or high, based on the 20th and 80th sex-specific percentiles of household income in the entire cancer population [18]. Education was grouped as low (elementary school), intermediate (high school) or high (university). A patient’s marital status was categorised as single (registered as not married, widow, divorced or separated) or married (registered as married or partner).

### Comorbidity

A patient’s co-existing diseases were measured using a modified version of the Charlson Comorbidity Index (CCI) using diagnostic codes (ICD-10) from hospitalisations within two years prior to, and including, the date of diagnosis [19, 20]. A score was determined for each of a patient’s recorded co-existing diseases based on its severity, and the combination of these scores resulted in a modified CCI. The CCI was grouped into “no hospital admissions”, low (CCI = 0), intermediate (CCI = 1,2) or high (CCI = 3+).

### Emergency presentation

The NPR registers the urgency of a patient’s visit to the hospital as either ‘acute’ or ‘planned’. A cancer diagnosis was defined as an ‘emergency presentation’ if an inpatient admittance with an acute urgency, irrespective of cause of admission, was registered in the NPR in the period from 30 days before to 2 days after the date of diagnosis.

### First/ever treatment

The first treatment was defined as the first registered occurrence of surgery of a primary tumour, radiotherapy (including chemoradiotherapy) or SACT (either alone or combined with radiotherapy) within one year of the date of diagnosis. Comprehensive information for surgery and radiotherapy was obtained from the CRN, while information on SACT was obtained from the NPR. SACT included chemotherapy, targeted therapy, immunotherapy and any other public hospital-administered anti-cancer medication. All medical procedure codes with the prefix WBOC and WML000 according to the Norwegian coding system for medical, surgical and radiological procedures were included [21]. Ever treatment referred to all treatment modalities a patient received within a year of diagnosis.

### Statistical analysis

Pearson’s chi-squared test was used to assess differences between the categories of the explanatory variables and a dichotomous variable indicating whether a patient was diagnosed following an emergency visit. Five multivariable logistic regressions were performed, with EP, surgery, radiotherapy, SACT and any treatment as the dependent variable, respectively [22]. These were all adjusted for case-mix, i.e., year of diagnosis, age group and stage at diagnosis, sex, region, income group, marital status, comorbidity index and cancer type. The analysis of receiving treatment (surgery, radiotherapy, SACT and any) was additionally adjusted for EP and CPP-status. Education was not adjusted for as the regressions would have given the marginal effects of both income and education, instead of the true effect of the patient’s SES. One-year overall survival was estimated for colorectal cancer patients, stratified by EP status, using the Kaplan-Meier method. Additionally, a multivariable Cox regression model adjusted for case-mix was performed to estimate the effect of EP on one-year overall survival. Multivariable quantile (median) regressions of waiting time from date of diagnosis to surgery, radiotherapy, SACT and any treatment were performed individually for all patients adjusted for case-mix and CPP-status. The regression analysing any treatment was additionally adjusted for treatment modality. Wald test was used to assess the significance of the different explanatory variables. Although colon and rectal cancer patients share the same CPP, their natural presentation, work-up and treatment differ, and therefore a stratified analysis was performed to examine differences between the two sites [23]. A *p*-value < 0.05 was considered significant. The statistical program Stata 16.1 was used for all analyses [24].

## Results

### Study population

Between 1 January 2015 and 31 December 2016, 8594 patients were identified with a primary colorectal cancer diagnosis. Patients registered solely by autopsy (*n* = 7) or death certificate (*n* = 96) were excluded. Also, patients under 18 years of age (n = 9), unknown place of residence (*n* = 53), unknown education (*n* = 58), unknown income (*n* = 1) and unknown type of household (*n* = 119) were excluded from the analyses. Finally, patients with a registered treatment prior to diagnosis were excluded (*n* = 35). As a result, 8216 patients were eligible for analyses.

### Patient characteristics

Of these 8216 patients diagnosed with colorectal cancer in 2015–2016 in Norway, 5677 (69.1%) had colon cancer and 2539 (30.9%) rectal cancer (Table 1). The proportion of diagnoses morphologically verified (either histologically or cytologically) was 97.6%. The proportion of males was 47.3% and the median age at diagnosis was 73 years [IQI: 65–81] for colon cancer, while the proportion of males was 59.6% and the median age at diagnosis was 69 years [IQI: 60–78] for rectal cancer. The proportions of patients who were diagnosed following an emergency visit were 35.4 and 15.5% for colon and rectal cancers, respectively (Supplementary Table 1, Supplementary Table 2). There were 1101 (19.4%) colon cancer patients and 244 (9.6%) rectal cancer patients whose date of resection was the same as their date of diagnosis.

**Table 1:** Patient characteristics, median waiting time with interquartile interval from date of diagnosis to treatment and the proportion treated with surgery, radiotherapy or systemic anticancer treatment, among colorectal cancer patients diagnosed in 2015–2016 in Norway

	All	Surgery			Radiotherapy			Systemic Anticancer Treatment		
	N (%)	N (%)	p50 [p25, p75]	% treated	N (%)	p50 [p25, p75]	% treated	N (%)	p50 [p25, p75]	% treated
Cancer type										
Colon	5677 (69.1%)	4672 (76.5%)	15.0 [3.0, 24.0]	82.3%	31 (4.5%)	34.0 [20.0, 53.0]	0.5%	449 (63.9%)	24.0 [15.0, 37.0]	7.9%
Rectal	2539 (30.9%)	1433 (23.5%)	25.0 [14.0, 35.0]	56.4%	664 (95.5%)	33.0 [27.0, 41.0]	26.2%	254 (36.1%)	27.0 [19.0, 38.0]	10.0%
Year of diagnosis										
2015	4114 (50.1%)	3074 (50.4%)	16.0 [4.0, 27.0]	74.7%	352 (50.6%)	34.0 [27.0, 41.0]	8.6%	328 (46.7%)	23.0 [16.0, 36.0]	8.0%
2016	4102 (49.9%)	3031 (49.6%)	17.0 [6.0, 27.0]	73.9%	343 (49.4%)	32.0 [26.0, 40.0]	8.4%	375 (53.3%)	26.0 [18.0, 38.0]	9.1%
Age group										
18–49	485 (5.9%)	343 (5.6%)	11.0 [0.0, 22.0]	70.7%	56 (8.1%)	29.5 [24.5, 38.0]	11.5%	60 (8.5%)	20.5 [17.0, 27.0]	12.4%
50–59	904 (11.0%)	609 (10.0%)	16.0 [2.0, 28.0]	67.4%	131 (18.8%)	33.0 [26.0, 40.0]	14.5%	135 (19.2%)	23.0 [16.0, 32.0]	14.9%
60–69	2098 (25.5%)	1535 (25.1%)	17.0 [4.0, 28.0]	73.2%	222 (31.9%)	33.0 [27.0, 42.0]	10.6%	258 (36.7%)	25.0 [17.0, 38.0]	12.3%
70–79	2629 (32.0%)	2095 (34.3%)	17.0 [8.0, 27.0]	79.7%	169 (24.3%)	34.0 [28.0, 42.0]	6.4%	209 (29.7%)	28.0 [19.0, 41.0]	7.9%
80–89	1790 (21.8%)	1361 (22.3%)	17.0 [7.0, 27.0]	76.0%	99 (14.2%)	33.0 [27.0, 41.0]	5.5%	41 (5.8%)	26.0 [15.0, 47.0]	2.3%
90+	310 (3.8%)	162 (2.7%)	12.0 [0.0, 25.0]	52.3%	18 (2.6%)	30.5 [23.0, 38.0]	5.8%	0 (0.0%)	–	0.0%
Sex										
Female	4018 (48.9%)	3069 (50.3%)	15.0 [4.0, 26.0]	76.4%	282 (40.6%)	33.5 [26.0, 40.0]	7.0%	296 (42.1%)	24.0 [15.0, 38.5]	7.4%
Male	4198 (51.1%)	3036 (49.7%)	18.0 [6.0, 28.0]	72.3%	413 (59.4%)	33.0 [27.0, 41.0]	9.8%	407 (57.9%)	25.0 [18.0, 36.0]	9.7%
Stage										
Localised	1767 (21.5%)	1670 (27.4%)	16.0 [0.0, 29.0]	94.5%	46 (6.6%)	32.0 [24.0, 38.0]	2.6%	5 (0.7%)	41.0 [17.0, 62.0]	0.3%
Regional	4118 (50.1%)	3587 (58.8%)	17.0 [9.0, 27.0]	87.1%	393 (56.5%)	33.0 [27.0, 40.0]	9.5%	74 (10.5%)	26.0 [17.0, 37.0]	1.8%
Metastasis	1716 (20.9%)	821 (13.4%)	13.0 [1.0, 24.0]	47.8%	143 (20.6%)	34.0 [25.0, 45.0]	8.3%	463 (65.9%)	25.0 [16.0, 36.0]	27.0%
Unknown	615 (7.5%)	27 (0.4%)	0.0 [0.0, 132.0]	4.4%	113 (16.3%)	34.0 [27.0, 42.0]	18.4%	161 (22.9%)	26.0 [18.0, 38.0]	26.2%
Marital status										
Single	3650 (44.4%)	2655 (43.5%)	16.0 [3.0, 27.0]	72.7%	298 (42.9%)	34.0 [26.0, 41.0]	8.2%	262 (37.3%)	26.5 [19.0, 40.0]	7.2%
Married	4566 (55.6%)	3450 (56.5%)	17.0 [7.0, 27.0]	75.6%	397 (57.1%)	33.0 [27.0, 40.0]	8.7%	441 (62.7%)	24.0 [16.0, 35.0]	9.7%
Income										
Low	1106 (13.5%)	816 (13.4%)	15.0 [3.0, 27.0]	73.8%	84 (12.1%)	31.0 [26.5, 38.5]	7.6%	77 (11.0%)	27.0 [17.0, 39.0]	7.0%
Intermediate	5155 (62.7%)	3846 (63.0%)	17.0 [6.0, 27.0]	74.6%	422 (60.7%)	34.0 [27.0, 42.0]	8.2%	389 (55.3%)	26.0 [18.0, 39.0]	7.5%
High	1955 (23.8%)	1443 (23.6%)	16.0 [4.0, 28.0]	73.8%	189 (27.2%)	33.0 [25.0, 39.0]	9.7%	237 (33.7%)	24.0 [15.0, 34.0]	12.1%
Education										
Low	2503 (30.5%)	1830 (30.0%)	16.0 [5.0, 27.0]	73.1%	198 (28.5%)	33.0 [27.0, 41.0]	7.9%	175 (24.9%)	25.0 [18.0, 38.0]	7.0%
Intermediate	3926 (47.8%)	2899 (47.5%)	17.0 [6.0, 27.0]	73.8%	370 (53.2%)	34.0 [27.0, 42.0]	9.4%	329 (46.8%)	25.0 [17.0, 37.0]	8.4%
High	1787 (21.8%)	1376 (22.5%)	16.0 [3.0, 27.0]	77.0%	127 (18.3%)	31.0 [22.0, 39.0]	7.1%	199 (28.3%)	25.0 [15.0, 38.0]	11.1%
Region										
Southern and Eastern Norway	4411 (53.7%)	3349 (54.9%)	17.0 [5.0, 27.0]	75.9%	334 (48.1%)	37.0 [30.0, 47.0]	7.6%	360 (51.2%)	27.0 [18.0, 39.0]	8.2%
Western Norway	1750 (21.3%)	1229 (20.1%)	16.0 [5.0, 28.0]	70.2%	155 (22.3%)	31.0 [26.0, 37.0]	8.9%	200 (28.4%)	21.0 [14.0, 33.5]	11.4%
Central Norway	1220 (14.8%)	915 (15.0%)	15.0 [6.0, 25.0]	75.0%	116 (16.7%)	26.5 [19.0, 34.5]	9.5%	90 (12.8%)	23.0 [15.0, 33.0]	7.4%
Northern Norway	835 (10.2%)	612 (10.0%)	17.0 [4.0, 30.0]	73.3%	90 (12.9%)	32.5 [27.0, 41.0]	10.8%	53 (7.5%)	30.0 [20.0, 47.0]	6.3%
Comorbidity										
No admissions	309 (3.8%)	230 (3.8%)	22.0 [12.0, 33.0]	74.4%	31 (4.5%)	39.0 [31.0, 55.0]	10.0%	35 (5.0%)	30.0 [21.0, 43.0]	11.3%
0	5926 (72.1%)	4395 (72.0%)	16.0 [6.0, 27.0]	74.2%	542 (78.0%)	33.0 [27.0, 40.0]	9.1%	555 (78.9%)	25.0 [17.0, 36.0]	9.4%
1–2	1671 (20.3%)	1266 (20.7%)	15.0 [3.0, 27.0]	75.8%	105 (15.1%)	32.0 [26.0, 43.0]	6.3%	108 (15.4%)	25.0 [16.0, 41.0]	6.5%
3+	310 (3.8%)	214 (3.5%)	17.0 [3.0, 28.0]	69.0%	17 (2.4%)	31.0 [27.0, 40.0]	5.5%	5 (0.7%)	34.0 [33.0, 44.0]	1.6%
Emergency Presentation										
No	5813 (70.8%)	4455 (73.0%)	19.0 [11.0, 29.0]	76.6%	583 (83.9%)	34.0 [27.0, 41.0]	10.0%	450 (64.0%)	27.0 [19.0, 39.0]	7.7%
Yes	2403 (29.2%)	1650 (27.0%)	7.0 [0.0, 19.0]	68.7%	112 (16.1%)	29.5 [21.0, 38.5]	4.7%	253 (36.0%)	21.0 [14.0, 34.0]	10.5%
CPP										
No	1519 (18.5%)	1043 (17.1%)	1.0 [0.0, 18.0]	68.7%	40 (5.8%)	32.5 [24.0, 41.5]	2.6%	132 (18.8%)	21.0 [14.0, 34.0]	8.7%
Yes	6697 (81.5%)	5062 (82.9%)	18.0 [10.0, 28.0]	75.6%	655 (94.2%)	33.0 [27.0, 41.0]	9.8%	571 (81.2%)	26.0 [18.0, 38.0]	8.5%

**Fig. 1:**
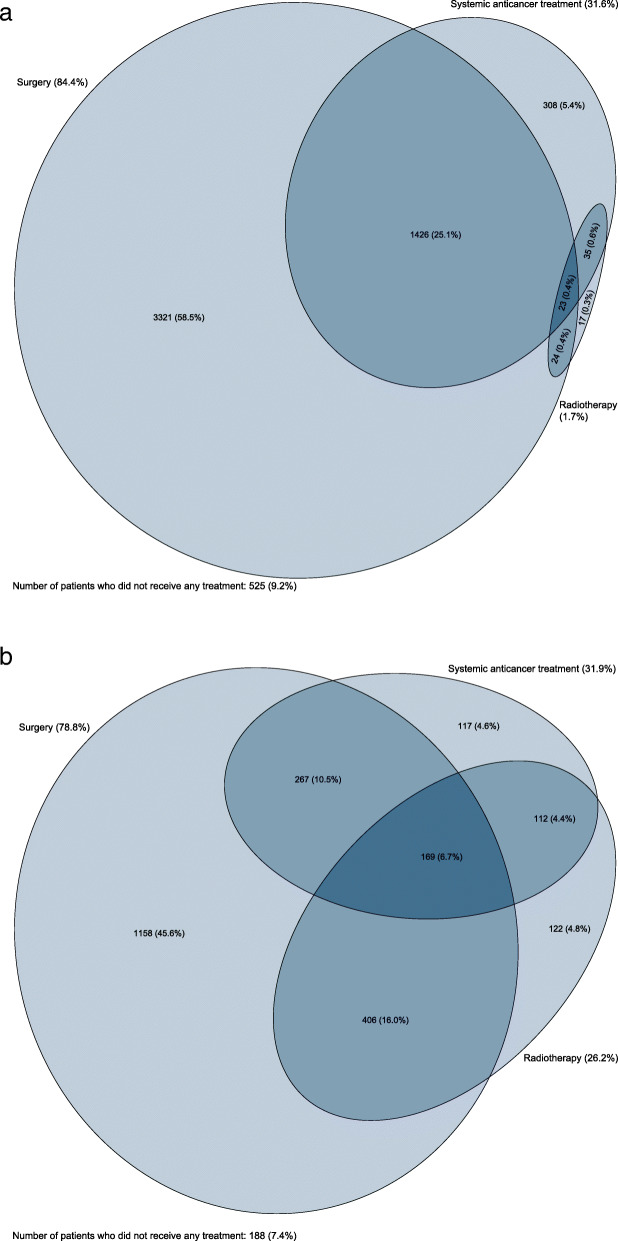
Euler diagram shows the combination of treatments received within one year of diagnosis among (**a**) colon cancer patients and (**b**) rectal cancer patients diagnosed in 2015–2016 in Norway

The proportions of colon cancer patients treated with surgery, radiotherapy or SACT within 12 months of diagnosis were 84.4, 1.7 and 31.6%, respectively (Fig. 1a). For colon cancer patients the first treatment was surgery for 82.3% and SACT for 7.9%. In addition, there were 31 colon cancer patients (0.5%) initially treated with radiotherapy, but these were excluded from the remaining analyses due to low numbers. Within one year of diagnosis, 525 (9.2%) colon cancer patients did not receive any form of tumour-directed treatment. The median age of this group was 82 years with the interquartile interval of 70–88 years. Of these, 357 patients (68.0%) died, and 168 patients (32.0%) were alive after one year, but remained untreated.

The proportions of rectal cancer patients treated with surgery, radiotherapy or SACT within 12 months of diagnosis were 78.8, 31.9 and 26.2%, respectively (Fig. 1b). For rectal cancer patients the first treatment was surgery for 56.4%, radiotherapy for 26.2% and SACT for 10.0%. Within one year of diagnosis, 188 (7.4%) rectal cancer patients did not receive any form of treatment. The median age of this group was 74 years with the interquartile interval of 60–85 years. Of these, 111 patients (59.0%) died, and 77 patients (41.0%) were alive without registered cancer treatment after one year.

### Emergency presentation

Among both colon and rectal cancer patients, older age, more advanced stage and higher level of comorbidity were significantly associated with increasing odds of being diagnosed during an EP (*p* < 0.001). Colorectal cancer patients with a regional (OR = 2.26, 95%CI: 1.93, 2.65) or metastatic (OR = 5.21, 95%CI: 4.38, 6.20) stage had over a 2- and 5-fold increased odds, respectively, of being diagnosed following an emergency visit compared to patients with a localised disease (Table 2). High-income patients had a 32% reduced odds of EP (OR = 0.68, 95%CI: 0.56, 0.82) as opposed to low-income patients. Patients who were married had a 28% reduced odds of an emergency diagnosis (OR = 0.72, 95%CI: 0.64, 0.80) compared to single patients. Colon cancer patients had a 2.7-fold increased odds of EP (OR = 2.66, 95%CI: 2.34, 3.02) compared to rectum cancer patients.

**Table 2:** Univariable and multivariable analysis of having an emergency presentation prior to diagnosis among colorectal cancer patients in 2015–2016 in Norway

	Univariate	Multivariate
	Odds Ratio [95%CI]	Odds Ratio [95%CI]
Year of diagnosis		
2015	1.00 (ref)	1.00 (ref)
2016	0.95 [0.86,1.04]	0.96 [0.86,1.06]
p-value	0.282	0.404
Age group		
18–49	1.14 [0.87,1.50]	1.19 [0.89,1.58]
50–59	1.00 (ref)	1.00 (ref)
60–69	1.19 [0.98,1.44]	1.07 [0.87,1.32]
70–79	1.59 [1.32,1.91]	1.23 [1.00,1.50]
80–89	2.56 [2.12,3.09]	1.75 [1.42,2.17]
90+	4.89 [3.71,6.46]	2.77 [2.04,3.77]
p-value	< 0.001	< 0.001
Sex		
Female	1.00 (ref)	1.00 (ref)
Male	0.81 [0.74,0.89]	0.96 [0.86,1.06]
p-value	< 0.001	0.416
Stage		
Localised	1.00 (ref)	1.00 (ref)
Regional	2.44 [2.10,2.84]	2.26 [1.93,2.65]
Metastasis	5.08 [4.30,5.99]	5.24 [4.40,6.23]
Unknown	3.64 [2.94,4.51]	3.37 [2.67,4.24]
p-value	< 0.001	< 0.001
Marital status		
Single	1.00 (ref)	1.00 (ref)
Married	0.65 [0.59,0.72]	0.72 [0.65,0.80]
p-value	< 0.001	< 0.001
Income		
Low	1.00 (ref)	1.00 (ref)
Intermediate	0.82 [0.71,0.93]	0.84 [0.72,0.97]
High	0.48 [0.41,0.57]	0.68 [0.57,0.82]
p-value	< 0.001	< 0.001
Region		
Southern and Eastern Norway	0.98 [0.93,1.02]	0.99 [0.95,1.04]
Western Norway	1.03 [0.94,1.12]	1.02 [0.92,1.13]
Central Norway	1.05 [0.94,1.18]	1.03 [0.91,1.16]
Northern Norway	1.00 [0.87,1.15]	0.96 [0.82,1.12]
p-value	0.708	0.905
Comorbidity		
No admissions	0.16 [0.09,0.26]	0.18 [0.11,0.30]
0	1.00 (ref)	1.00 (ref)
1–2	1.92 [1.71,2.15]	1.74 [1.54,1.97]
3+	3.49 [2.77,4.40]	3.15 [2.44,4.06]
p-value	< 0.001	< 0.001
Cancer type		
Rectum	1.00 (ref)	1.00 (ref)
Colon	2.97 [2.64,3.35]	2.66 [2.34,3.02]
p-value	< 0.001	< 0.001

A total of 2403 colorectal cancer patients were diagnosed after an emergency presentation. The cause of admission was registered as “Malignant tumour in the colon, rectosigmoid or rectum” (70.3%), “Diseases of the digestive system” (8.0%), “Benign or unknown malignancy tumours – any location” (6.9%), “Other malignant tumours than C18–20” (4.2%), “Deficiency anaemia, haemolytic anaemia, aplastic anaemia and other anaemia” (4.2%) and “Others” (6.5%).” The median time from admission to hospital to cancer diagnosis ranged from 2 days (IQI: 0–4) in “Malignant tumour in the colon, rectosigmoid or rectum” to 7 days (IQI: 4–15) in “Deficiency anaemia, haemolytic anaemia, aplastic anaemia and other anaemia.

One-year overall survival among patients who had an emergency presentation prior to a colorectal cancer diagnosis was 67.7% (95%CI: 65.8–69.6%) while it was 90.2% (95%CI: 89.4–90.9%) for patients without an emergency presentation. After adjusting for case-mix, patients with an emergency presentation were 87% (HR = 1.87, 95%CI: 1.72–2.02) more likely to die within one year of diagnosis compared to patients without an emergency presentation (data not shown).

### Waiting time to treatment

The median waiting time for colorectal cancer patients to any treatment increased with increasing age, and patients aged 80–89 years waited three days longer than patients aged 18–49 years (Table 3). Colorectal cancer patients with a regional disease had a median 2.3-day (95%CI: 1.4, 3.0) longer waiting time to surgery, compared to localised patients (Table 3). Colorectal cancer patients with an EP had a 7.0-day (95%CI: − 7.9, − 6.1) shorter median waiting time to any treatment compared to those with a non-EP.

**Table 3:** Univariable and multivariable analyses of median time from diagnosis to first treatment (surgery, radiotherapy, systemic anticancer treatment or any treatment) among colorectal cancer patients diagnosed in 2015–2016 in Norway

	Surgery		Radiotherapy		Systemic Anticancer Treatment		Any	
	Univariate	Multivariate	Univariate	Multivariate	Univariate	Multivariate	Univariate	Multivariate
	Coeff (95%CI)	Coeff (95%CI)	Coeff (95%CI)	Coeff (95%CI)	Coeff (95%CI)	Coeff (95%CI)	Coeff (95%CI)	Coeff (95%CI)
Year of diagnosis								
2015	0.00 (ref)	0.00 (ref)	0.00 (ref)	0.00 (ref)	0.00 (ref)	0.00 (ref)	0.00 (ref)	0.00 (ref)
2016	1.00 [−0.06,2.06]	0.13 [− 0.66,0.92]	− 2.00 [−3.88,-0.12]	−2.00 [−3.82,-0.18]	3.00 [0.28,5.72]	2.88 [− 0.04,5.79]	0.00 [− 0.80,0.80]	0.29 [− 0.43,1.00]
p-value	0.065	0.750	0.037	0.032	0.030	0.053	1.00	0.431
Age group								
18–49	−5.00 [−7.82,-2.18]	−2.79 [−4.88,-0.71]	−3.00 [−6.83,0.83]	−3.00 [−6.79,0.79]	−2.00 [− 7.95,3.95]	−1.13 [−6.98,4.73]	−5.00 [− 7.12,-2.88]	−2.57 [− 4.35,-0.80]
50–59	0.00 (ref)	0.00 (ref)	0.00 (ref)	0.00 (ref)	0.00 (ref)	0.00 (ref)	0.00 (ref)	0.00 (ref)
60–69	1.00 [−1.00,3.00]	1.08 [−0.40,2.56]	0.00 [−2.64,2.64]	2.00 [− 0.68,4.68]	2.00 [− 2.08,6.08]	2.87 [− 1.13,6.88]	−1.00 [− 2.49,0.49]	1.43 [0.17,2.68]
70–79	1.00 [− 0.92,2.92]	1.90 [0.42,3.37]	1.00 [−1.80,3.80]	2.00 [− 1.00,5.00]	5.00 [0.76,9.24]	5.00 [0.64,9.36]	−1.00 [− 2.45,0.45]	2.43 [1.16,3.70]
80–89	1.00 [−1.03,3.03]	2.56 [0.99,4.14]	0.00 [−3.23,3.23]	3.00 [−0.43,6.43]	3.00 [− 3.84,9.84]	2.88 [− 3.96,9.71]	−3.00 [− 4.56,-1.44]	3.00 [1.61,4.39]
90+	− 4.00 [−7.69,-0.31]	1.33 [− 1.45,4.12]	1.00 [−5.25,7.25]	−1.00 [− 7.37,5.37]	–	–	− 7.00 [− 10.02,-3.98]	1.00 [− 1.59,3.59]
p-value	< 0.001	< 0.001	0.469	0.064	0.066	0.131	< 0.001	< 0.001
Sex								
Female	0.00 (ref)	0.00 (ref)	0.00 (ref)	0.00 (ref)	0.00 (ref)	0.00 (ref)	0.00 (ref)	0.00 (ref)
Male	3.00 [1.94,4.06]	0.69 [−0.12,1.50]	−1.00 [−2.96,0.96]	− 1.00 [− 2.87,0.87]	1.00 [− 1.69,3.69]	0.25 [− 2.67,3.17]	2.00 [1.09,2.91]	0.71 [− 0.02,1.44]
*p*-value	< 0.001	0.094	0.316	0.295	0.466	0.867	< 0.001	0.055
Stage								
Localised	0.00 (ref)	0.00 (ref)	0.00 (ref)	0.00 (ref)	0.00 (ref)	0.00 (ref)	0.00 (ref)	0.00 (ref)
Regional	1.00 [−0.16,2.16]	2.44 [1.50,3.38]	1.00 [−2.66,4.66]	1.50 [−2.25,5.25]	−15.00 [− 31.92,1.92]	− 16.13 [− 33.52,1.27]	2.00 [0.81,3.19]	2.29 [1.38,3.20]
Metastasis	− 3.00 [−4.67,-1.33]	0.59 [− 0.76,1.94]	3.00 [− 1.05,7.05]	4.50 [0.39,8.61]	−16.00 [− 32.46,0.46]	−17.63 [− 34.57,-0.68]	2.00 [0.51,3.49]	0.57 [− 0.67,1.81]
Unknown	− 16.00 [− 23.61,-8.39]	− 0.90 [−6.84,5.05]	2.00 [− 2.12,6.12]	4.00 [− 0.19,8.19]	−15.00 [− 31.62,1.62]	− 15.38 [− 32.52,1.77]	12.00 [9.40,14.60]	1.57 [− 0.60,3.74]
*p*-value	< 0.001	< 0.001	0.307	0.022	0.268	0.132	< 0.001	< 0.001
Marital status								
Single	0.00 (ref)	0.00 (ref)	0.00 (ref)	0.00 (ref)	0.00 (ref)	0.00 (ref)	0.00 (ref)	0.00 (ref)
Married	1.00 [0.05,1.95]	−0.56 [− 1.40,0.27]	−2.00 [−3.68,-0.32]	− 1.00 [− 2.90,0.90]	−3.00 [−6.19,0.19]	−3.37 [− 6.34,-0.41]	1.00 [0.08,1.92]	−0.86 [− 1.61,-0.11]
p-value	0.040	0.185	0.020	0.303	0.066	0.026	0.034	0.025
Income								
Low	0.00 (ref)	0.00 (ref)	0.00 (ref)	0.00 (ref)	0.00 (ref)	0.00 (ref)	0.00 (ref)	0.00 (ref)
Intermediate	2.00 [0.71,3.29]	0.82 [−0.38,2.02]	3.00 [−0.17,6.17]	1.00 [−1.89,3.89]	−1.00 [− 5.54,3.54]	− 0.50 [− 5.17,4.17]	2.00 [0.82,3.18]	0.86 [− 0.24,1.95]
High	1.00 [− 0.47,2.47]	0.28 [− 1.15,1.71]	2.00 [− 1.47,5.47]	0.00 [− 3.33,3.33]	−3.00 [− 7.77,1.77]	− 3.25 [− 8.32,1.82]	1.00 [− 0.32,2.32]	0.00 [− 1.29,1.29]
p-value	0.004	0.296	0.163	0.611	0.312	0.217	0.001	0.087
Region								
Southern and Eastern Norway	0.75 [−0.01,1.51]	0.13 [− 0.48,0.73]	5.25 [3.91,6.59]	5.88 [4.48,7.27]	1.75 [−0.88,4.38]	1.16 [− 1.16,3.47]	0.50 [− 0.16,1.16]	0.82 [0.27,1.37]
Western Norway	−0.25 [−1.22,0.72]	− 0.54 [− 1.32,0.24]	−0.75 [− 2.38,0.88]	−1.13 [− 2.82,0.57]	−4.25 [− 7.25,-1.25]	−3.22 [− 5.86,-0.58]	−0.50 [− 1.34,0.34]	−0.75 [− 1.44,-0.06]
Central Norway	−1.25 [− 2.32,-0.18]	−0.74 [− 1.60,0.12]	−5.75 [− 7.56,-3.94]	−5.63 [− 7.49,-3.76]	−2.25 [− 6.07,1.57]	−3.22 [− 6.63,0.19]	− 1.50 [− 2.44,-0.56]	−1.32 [− 2.10,-0.55]
Northern Norway	0.75 [−0.49,1.99]	1.15 [0.15,2.15]	1.25 [−0.72,3.22]	0.88 [−1.18,2.93]	4.75 [0.08,9.42]	5.28 [1.14,9.43]	1.50 [0.42,2.58]	1.25 [0.35,2.15]
p-value	0.032	0.071	< 0.001	< 0.001	0.004	0.005	0.001	< 0.001
Emergency Presentation								
0	0.00 (ref)	0.00 (ref)	0.00 (ref)	0.00 (ref)	0.00 (ref)	0.00 (ref)	0.00 (ref)	0.00 (ref)
1	− 12.00 [− 13.13,-10.87]	−7.00 [− 7.95,-6.05]	−2.00 [− 4.49,0.49]	−3.00 [−5.69,-0.31]	−6.00 [− 8.92,-3.08]	− 5.75 [− 8.88,-2.62]	− 10.00 [− 10.84,-9.16]	−7.00 [− 7.86,-6.14]
p-value	< 0.001	< 0.001	0.116	0.029	< 0.001	< 0.001	< 0.001	< 0.001
Comorbidity								
No admissions	6.00 [3.48,8.52]	4.67 [2.58,6.76]	3.00 [− 1.44,7.44]	8.00 [3.55,12.45]	5.00 [−1.14,11.14]	5.63 [−0.98,12.23]	7.00 [4.50,9.50]	4.29 [2.44,6.13]
0	0.00 (ref)	0.00 (ref)	0.00 (ref)	0.00 (ref)	0.00 (ref)	0.00 (ref)	0.00 (ref)	0.00 (ref)
1–2	−1.00 [−2.19,0.19]	−0.03 [− 1.03,0.98]	−1.00 [−3.54,1.54]	− 0.50 [− 3.14,2.14]	1.00 [− 2.71,4.71]	2.00 [− 2.03,6.03]	−1.00 [− 2.22,0.22]	−0.29 [− 1.21,0.63]
3+	1.00 [− 1.60,3.60]	1.97 [− 0.20,4.15]	0.00 [−5.91,5.91]	− 2.00 [−7.97,3.97]	9.00 [−6.83,24.83]	7.13 [−9.75,24.00]	1.00 [− 1.78,3.78]	1.71 [− 0.35,3.78]
p-value	< 0.001	< 0.001	0.464	0.004	0.281	0.258	< 0.001	< 0.001
Cancer type								
Rectum	0.00 (ref)	0.00 (ref)			0.00 (ref)	0.00 (ref)	0.00 (ref)	0.00 (ref)
Colon	−10.00 [−10.97,-9.03]	−8.54 [−9.50,-7.57]			−3.00 [−5.69,-0.31]	−1.87 [− 5.03,1.28]	−13.00 [− 13.66,-12.34]	−7.43 [− 8.32,-6.53]
p-value	< 0.001	< 0.001			0.029	0.243	< 0.001	< 0.001
CPP								
0	0.00 (ref)	0.00 (ref)	0.00 (ref)	0.00 (ref)	0.00 (ref)	0.00 (ref)	0.00 (ref)	0.00 (ref)
1	17.00 [15.85,18.15]	9.77 [8.69,10.85]	0.00 [−4.13,4.13]	−3.00 [−7.31,1.31]	5.00 [1.61,8.39]	2.12 [−1.65,5.90]	17.00 [15.87,18.13]	9.00 [8.00,10.00]
p-value	< 0.001	< 0.001	1.00	0.172	0.004	0.269	< 0.001	< 0.001
Treatment								
Surgery							0.00 (ref)	0.00 (ref)
Radiotherapy							17.00 [15.46,18.54]	9.14 [7.66,10.62]
Chemotherapy							9.00 [7.50,10.50]	9.86 [8.38,11.34]
p-value							< 0.001	< 0.001

In addition to the results above for colon and rectal patients, the time to surgery and SACT for colon cancer patients increased with increasing level of comorbidity (Supplementary Table 3). Married colon cancer patients had a 6.0-day (95%CI: − 10.3, − 1.7) shorter time to SACT compared to single patients. Colon cancer patients who had an EP had a 7.1-day (95%CI: − 8.1, − 6.2) shorter median waiting time to any treatment compared to patients with no EP. The waiting time to SACT as compared to surgery, was almost 14 days longer (Supplementary Table 3).

Among rectal cancer, male patients experienced a 3.3-day shorter time to surgery compared to females (95%CI: 0.8, 5.7). Being married was associated with a 3-day (Coeff: -3.1, 95%CI: − 5.6, − 0.7) shorter waiting time to surgery among rectal cancer patients (Supplementary Table 4). The waiting time to radiotherapy among rectal cancer patients, was almost seven days longer than the waiting time to surgery (Supplementary Table 4). Rectal cancer patients living in Western Norway and Northern Norway had a 5.8-day (95%CI: − 9.7, − 1.9) shorter and 9.5-day (95%CI: 3.4, 15.6) longer waiting time, respectively, to SACT compared to the national median, and patients living in Central Norway had a 2.9-day (95%CI: − 5.4, − 0.3) shorter and 5.6-day (95%CI: − 7.5, − 3.8) shorter waiting time to surgery and radiotherapy, respectively. Rectal cancer patients living in Southern and Eastern Norway, experienced a 5.9-day (95%CI: 4.5, 7.3) longer waiting time to radiotherapy than the national median.

### Access to first treatment modalities

Treatment modality varied by cancer type and stage of disease (Table 1, Supplementary Table 1, Supplementary Table 2). Table 4 shows that that the odds of receiving SACT as the first treatment decreased with increasing age and more comorbidities (*p* < 0.001) among colorectal cancer patients. The odds of receiving surgery was lower for metastatic colorectal cancer patients (OR = 0.02, 95%CI: 0.02, 0.03). Compared to Norway, patients living in Western Norway had a 59% increased odds (OR = 1.59, 95%CI: 1.35, 1.88) of SACT and patients from Northern Norway had a 36% reduced odds (OR = 0.64, 95%CI: 0.47, 0.85) of SACT (Table 4).

**Table 4:** Univariable and multivariable analyses for having surgery, radiotherapy, systemic anticancer treatment or any treatment among colorectal cancer patients diagnosed in 2015–2016 in Norway

	Surgery		Radiotherapy		Systemic Anticancer Treatment		Any	
	Univariate	Multivariate	Univariate	Multivariate	Univariate	Multivariate	Univariate	Multivariate
	Odds Ratio (95%CI)	Odds Ratio (95%CI)	Odds Ratio (95%CI)	Odds Ratio (95%CI)	Odds Ratio (95%CI)	Odds Ratio (95%CI)	Odds Ratio (95%CI)	Odds Ratio (95%CI)
**Year of diagnosis**								
2015	1.00 (ref)	1.00 (ref)	1.00 (ref)	1.00 (ref)	1.00 (ref)	1.00 (ref)	1.00 (ref)	1.00 (ref)
2016	0.96 [0.87,1.06]	0.95 [0.83,1.09]	0.97 [0.83,1.14]	0.87 [0.72,1.05]	1.16 [1.00,1.36]	1.03 [0.86,1.24]	1.03 [0.91,1.15]	1.02 [0.89,1.18]
*p*-value	0.398	0.453	0.717	0.142	0.058	0.714	0.662	0.776
**Age group**								
18–49	1.17 [0.92,1.49]	1.20 [0.87,1.65]	0.77 [0.55,1.08]	0.84 [0.56,1.26]	0.80 [0.58,1.11]	0.83 [0.56,1.21]	1.06 [0.79,1.42]	1.05 [0.73,1.50]
50–59	1.00 (ref)	1.00 (ref)	1.00 (ref)	1.00 (ref)	1.00 (ref)	1.00 (ref)	1.00 (ref)	1.00 (ref)
60–69	1.32 [1.11,1.56]	1.19 [0.95,1.49]	0.70 [0.55,0.88]	0.79 [0.59,1.05]	0.80 [0.64,1.00]	0.91 [0.69,1.19]	1.26 [1.02,1.56]	1.13 [0.88,1.46]
70–79	1.91 [1.61,2.26]	1.71 [1.35,2.16]	0.40 [0.31,0.51]	0.49 [0.36,0.67]	0.49 [0.39,0.62]	0.59 [0.44,0.78]	1.53 [1.24,1.88]	1.24 [0.95,1.61]
80–89	1.54 [1.29,1.84]	1.40 [1.09,1.80]	0.33 [0.25,0.44]	0.49 [0.34,0.69]	0.13 [0.09,0.19]	0.13 [0.09,0.20]	0.77 [0.63,0.95]	0.55 [0.42,0.72]
90+	0.53 [0.41,0.69]	0.59 [0.40,0.88]	0.33 [0.19,0.57]	0.55 [0.28,1.08]	1.00 [1.00,1.00]	1.00 [1.00,1.00]	0.23 [0.18,0.31]	0.20 [0.14,0.29]
*p*-value	<0.001	<0.001	<0.001	<0.001	<0.001	<0.001	<0.001	<0.001
**Sex**								
Female	1.00 (ref)	1.00 (ref)	1.00 (ref)	1.00 (ref)	1.00 (ref)	1.00 (ref)	1.00 (ref)	1.00 (ref)
Male	0.80 [0.73,0.89]	0.90 [0.79,1.04]	1.49 [1.26,1.75]	0.98 [0.81,1.19]	1.35 [1.15,1.58]	1.22 [1.01,1.47]	0.88 [0.78,0.99]	0.97 [0.84,1.13]
*p*-value	<0.001	0.159	<0.001	0.847	<0.001	0.036	0.028	0.706
**Stage**								
Localised	1.00 (ref)	1.00 (ref)	1.00 (ref)	1.00 (ref)	1.00 (ref)	1.00 (ref)	1.00 (ref)	1.00 (ref)
Regional	0.39 [0.31,0.48]	0.27 [0.21,0.35]	4.10 [2.98,5.62]	7.32 [5.23,10.23]	6.45 [2.60,15.97]	6.73 [2.71,16.70]	0.43 [0.34,0.55]	0.32 [0.25,0.41]
Metastasis	0.05 [0.04,0.07]	0.02 [0.02,0.03]	3.03 [2.13,4.31]	5.10 [3.50,7.44]	132.29 [54.64,320.28]	148.20 [60.96,360.32]	0.17 [0.13,0.21]	0.10 [0.08,0.14]
Unknown	0.00 [0.00,0.00]	0.00 [0.00,0.00]	8.41 [5.85,12.11]	9.46 [6.34,14.13]	126.22 [51.52,309.22]	205.52 [83.21,507.62]	0.02 [0.02,0.03]	0.02 [0.01,0.02]
*p*-value	<0.001	<0.001	<0.001	<0.001	<0.001	<0.001	<0.001	<0.001
**Marital status**								
Single	1.00 (ref)	1.00 (ref)	1.00 (ref)	1.00 (ref)	1.00 (ref)	1.00 (ref)	1.00 (ref)	1.00 (ref)
Married	1.16 [1.05,1.28]	1.04 [0.90,1.20]	1.09 [0.93,1.28]	1.00 [0.82,1.22]	1.38 [1.18,1.62]	1.23 [1.02,1.49]	1.45 [1.29,1.63]	1.20 [1.04,1.40]
*p*-value	0.005	0.613	0.297	0.982	<0.001	0.030	<0.001	0.016
**Income**								
Low	1.00 (ref)	1.00 (ref)	1.00 (ref)	1.00 (ref)	1.00 (ref)	1.00 (ref)	1.00 (ref)	1.00 (ref)
Intermediate	1.05 [0.90,1.21]	1.04 [0.85,1.29]	1.08 [0.84,1.39]	0.91 [0.67,1.22]	1.09 [0.85,1.41]	1.12 [0.84,1.50]	1.10 [0.93,1.30]	1.13 [0.91,1.39]
High	1.00 [0.84,1.18]	1.06 [0.83,1.35]	1.32 [1.01,1.74]	0.79 [0.56,1.11]	1.84 [1.41,2.41]	1.29 [0.93,1.78]	1.46 [1.20,1.78]	1.33 [1.03,1.72]
*p*-value	0.682	0.894	0.051	0.339	<0.001	0.261	<0.001	0.078
**Region**								
Southern and Eastern Norway	1.09 [1.04,1.14]	1.11 [1.04,1.18]	0.88 [0.82,0.95]	0.85 [0.77,0.93]	0.97 [0.90,1.04]	0.95 [0.88,1.04]	1.10 [1.04,1.16]	1.08 [1.01,1.15]
Western Norway	0.81 [0.73,0.88]	0.71 [0.63,0.80]	1.09 [0.94,1.27]	1.05 [0.88,1.25]	1.40 [1.22,1.60]	1.59 [1.35,1.88]	0.91 [0.81,1.01]	0.89 [0.78,1.02]
Central Norway	1.04 [0.92,1.17]	1.27 [1.07,1.52]	1.15 [0.96,1.38]	1.28 [1.03,1.60]	0.87 [0.71,1.05]	0.83 [0.66,1.04]	0.96 [0.84,1.11]	1.15 [0.97,1.37]
Northern Norway	0.94 [0.81,1.09]	0.85 [0.70,1.04]	1.35 [1.09,1.67]	1.51 [1.15,1.97]	0.73 [0.57,0.95]	0.64 [0.47,0.85]	0.80 [0.68,0.94]	0.69 [0.57,0.85]
*p*-value	<0.001	<0.001	0.002	<0.001	<0.001	<0.001	0.002	<0.001
**Emergency Presentation**								
0	1.00 (ref)	1.00 (ref)	1.00 (ref)	1.00 (ref)	1.00 (ref)	1.00 (ref)	1.00 (ref)	1.00 (ref)
1	0.67 [0.61,0.75]	0.75 [0.64,0.88]	0.39 [0.31,0.49]	0.96 [0.73,1.26]	1.41 [1.20,1.66]	1.10 [0.90,1.35]	0.71 [0.63,0.81]	0.76 [0.64,0.89]
*p*-value	<0.001	<0.001	<0.001	0.752	<0.001	0.345	<0.001	0.001
**Comorbidity**								
No admissions	1.02 [0.79,1.34]	1.26 [0.87,1.83]	1.08 [0.73,1.60]	0.73 [0.46,1.14]	1.24 [0.86,1.78]	1.24 [0.79,1.94]	1.21 [0.87,1.69]	1.52 [1.01,2.29]
0	1.00 (ref)	1.00 (ref)	1.00 (ref)	1.00 (ref)	1.00 (ref)	1.00 (ref)	1.00 (ref)	1.00 (ref)
1-2	1.09 [0.96,1.23]	1.15 [0.96,1.37]	0.66 [0.53,0.83]	0.97 [0.74,1.26]	0.67 [0.54,0.83]	0.72 [0.56,0.92]	0.91 [0.78,1.05]	0.96 [0.80,1.15]
3+	0.77 [0.60,0.99]	0.92 [0.64,1.33]	0.57 [0.34,0.94]	0.89 [0.48,1.63]	0.16 [0.07,0.39]	0.16 [0.06,0.40]	0.47 [0.36,0.60]	0.51 [0.37,0.71]
*p*-value	0.093	0.269	<0.001	0.566	<0.001	<0.001	<0.001	<0.001
**Cancer type**								
Rectum	1.00 (ref)	1.00 (ref)	1.00 (ref)	1.00 (ref)	1.00 (ref)	1.00 (ref)	1.00 (ref)	1.00 (ref)
Colon	3.70 [3.33,4.11]	9.29 [7.88,10.96]	1.00 [1.00,1.00]	1.00 [1.00,1.00]	0.78 [0.66,0.91]	0.96 [0.78,1.17]	4.93 [4.36,5.56]	9.67 [8.21,11.39]
*p*-value	<0.001	<0.001			0.002	0.672	<0.001	<0.001
**CPP**								
0	1.00 (ref)	1.00 (ref)	1.00 (ref)	1.00 (ref)	1.00 (ref)	1.00 (ref)	1.00 (ref)	1.00 (ref)
1	1.41 [1.24,1.59]	1.36 [1.14,1.62]	5.00 [3.47,7.20]	3.32 [2.22,4.97]	0.98 [0.80,1.19]	1.14 [0.90,1.45]	1.54 [1.34,1.77]	1.64 [1.37,1.97]
*p*-value	<0.001	<0.001	<0.001	<0.001	0.814	0.282	<0.001	<0.001

In addition, male and married colon cancer patients experienced a 30% (OR = 1.30, 95%CI: 1.03, 1.64) and 39% (OR = 1.39, 95%CI: 1.09, 1.77) increased odds of SACT (Supplementary Table 5). Compared to Norway, colon cancer patients living in Western Norway had a 44% reduced odds (OR = 0.56, 95%CI: 0.47, 0.67) of surgery.

Supplementary Fig. 1 and supplementary Table 6 show that that the odds of receiving radiotherapy as the first treatment decreased with increasing age (*p* < 0.001) among rectal cancer patients. The opposite pattern was observed for surgery, where there was an increased odds among patients aged 60–69 (OR = 1.48, 95%CI: 1.09, 2.01), 70–79 (OR = 2.79, 95%CI: 2.01, 3.88) and 80–89 (OR = 2.40, 95%CI: 1.66, 3.46) compared with patients aged 50–59 years. The odds of receiving surgery decreased with more advanced cancer (p < 0.001), while patients with a regional or metastatic disease experienced over a 7-fold (OR: 7.32, 95%CI: 5.23, 10.23) and 5-fold (OR: 5.10, 95%CI: 3.50, 7.44) increase in the odds of receiving radiotherapy, respectively. Rectal cancer patients with a high income had a 48% (OR = 1.48, 95%CI: 1.03, 2.13) increased odds of receiving surgery. Patients from Northern Norway had a 51% increased odds (OR = 1.51, 95%CI: 1.15, 1.97) of radiotherapy compared to Norway. Those patients who were included in a CPP experienced over a 3-fold increase in the odds of radiotherapy (OR = 3.32, 95%CI: 2.22, 4.97).

## Discussion

The odds of having an EP prior to a colon or rectal cancer diagnosis increased with increasing age, more advanced stage and a higher level of comorbidity, while the odds decreased for patients who were married or had a high income. Patients having an EP were more likely to die within the first year after diagnosis. The waiting time to any treatment increased with increasing age and stage for all colon and rectal cancer patients. Compared with the waiting time to surgery, longer times to SACT and radiotherapy were observed for colon and rectal cancers, respectively. Regional differences in waiting time to treatment and choice of treatment existed within both colon and rectal cancers. Access to treatment for colorectal cancer patients was associated with the patient’s age and stage of disease. Additionally, gender and marital status were associated with the likelihood of receiving SACT among colon cancer patients, while income was associated with surgery among rectal cancer patients.

The proportion of emergency-onset diagnosis among colon cancer patients was 35%, while it was 15% among rectal cancer patients. It is important to distinguish between EPs as part of the route to diagnosis and emergency resection at diagnosis. Earlier studies have shown that 15–25% of all colon cancer patients, and less than 5% of rectal cancer patients were hospitalised and resected at the time of diagnosis due to acute symptoms such as obstruction, perforation or bleeding [25]. The higher proportions observed in the present study may be due to the definition of EP, which included all registered hospital visits irrespective of cause of admission up to 30 days prior to a colorectal cancer diagnosis, and not limited to those resected at the time of diagnosis. For example, if a patient presents at a hospital for a non-colorectal cancer-related emergency, and then within 30 days has a colorectal cancer diagnosis, the patient would be registered as having a colorectal cancer EP. This definition should be considered when interpreting the results.

Similar to previous studies, this study showed that the proportion of EPs and waiting time and access to tumour-directed treatment were associated with both age and stage among colon and rectal cancer patients [6, 26]. Older patients may have more chronic diseases and hospital admissions than younger patients [20]. Therefore, older colorectal cancer patients may be more often admitted to hospital to have more complex diagnostic examinations rather than having these performed during an outpatient visit. Residual confounding in comorbidity may also partially explain the association between age, EP and waiting times for treatment. Although comorbidity was adjusted for through the grouped CCI, more detailed information about coexisting diseases could explain some of the age effect. Patients with more advanced stage of disease may experience more severe symptoms such as intestinal obstruction, anemia, and reduced general condition, which may increase the proportion of (emergency) hospital admissions [26]. Older patients with comorbidity may undergo more extensive examinations, such as a geriatric assessment, prior to treatment selection [27]. Disparities in treatment and survival occur between countries, for example, colon cancer patients over 80 years of age in the UK have a lower likelihood of resection and lower survival than in Norway [6, 28]. Studies have also shown that increased age was associated with an increased proportion of right-sided colon cancers, which have an increased risk of death compared to left-sided colon cancers [29]. Patients who had an EP prior to a diagnosis were found to be 87% more likely to die within one year of diagnosis. In addition to older age, more advanced stage and more comorbidities, EP patients often present with poorer performance status, and may have tumour blockage which can lead to subileus or ileus, or perforation resulting in abdominal infections. Patients with EP are more likely to have emergency surgery. These factors may additionally contribute to the lower survival.

It is reasonable to observe that patients with more advanced stage experience longer waiting times to treatment, as these patients may require a more extensive diagnostic period for complete staging and consideration of feasible treatment options. For example, colon cancer with regional spread may include anything from a local tumour with malignant lymph nodes, to tumours with perforation of the visceral peritoneum, to direct growth into other organs or structures. Additional diagnostic procedures may be necessary to determine resectability. Rectal cancer patients with regional disease are often referred to a regional hospital with a radiotherapy unit, and their treatment plan is discussed at a multidisciplinary team meeting. This may contribute to the longer waiting time to treatment experienced by more advanced stage rectal cancer patients.

This study found regional differences among colorectal cancer patients, both in waiting times and in access to treatment. Part of the difference may be explained by geographical distance between regions. During the study period, patient referrals were sent to the hospitals via postal service, and handling of referrals may have varied between hospitals. Despite having national guidelines for diagnosis and treatment of colorectal cancer, the use of radiotherapy among rectal cancer patients varied between different regions in Norway. The 2019 annual report from the Norwegian Colorectal Cancer Registry showed that the use of preoperative radiotherapy ranged from 30.1 to 39.6% between the different health regions in Norway [30]. Similar results have been reported from the UK [31]. Reasons for observed regional variation in radiotherapy use may be due to the uncertainties in evaluating pathological lymph nodes with an MRI, and differing practices regarding treatment with radiotherapy when the lymph nodes are uncertain. As the guidelines across Europe do not have a common optimal level of preoperative radiotherapy, some variation across regions is expected [32, 33]. In addition, this study also found that the use of SACT among colon cancer patients varied significantly between regions, which may also indicate different local practice and individual interpretation of the national guidelines. It is also possible that inclusion in a clinical phase III study at Western Norway, comparing addition of neoadjuvant chemotherapy with standard treatment in patients with locally advanced colon cancer, may explain some of the increased odds of receiving SACT.

Rectal cancer patients had over a week longer waiting time to start radiotherapy than to surgery, although the CPP guidelines state that radiotherapy should start within 39 days of start CPP, i.e., four days longer than time to surgery. Radiotherapy treatment in Norway is centralised to fewer hospitals than surgical treatment. Therefore, the referral time from local hospital to a radiotherapy centre may in part explain the increased time among rectal cancer patients who are initially treated with radiotherapy. Although this study defines start of radiotherapy as the first day the patient received treatment, the period prior to treatment is dedicated to treatment planning. Thus, it may be more appropriate to use the phrase “time to start radiotherapy” rather than “waiting time”.

Married and high-income colorectal cancer patients were less likely to enter the hospital as an EP, while no effect was seen in waiting times. These results are consistent with the general understanding that health awareness and lifestyle are superior among high SES patients compared with low. From these results, patients’ SES seems to affect how quickly patients were seeking help, but after entering the health care system, no systematic differences existed in how quickly patients were treated. Similar to what earlier studies found, high-income rectal cancer patients had a significantly increased odds of treatment compared with low-income patients [13, 34]. In Norway, there are several private providers of colonoscopy services where waiting times may be shorter than at public hospitals. Use of these private services may be more common among high-income patients, which may potentially enable them to be diagnosed earlier and therefore be more eligible for resection. Earlier detection may result in better surgery results. For example, a Swedish study found that high SES rectal cancer patients had a lower proportion of surgeries resulting in colostomy [12].

This study has some limitations. It may be possible that the proportion of patients with an EP was overestimated, however, by using as many as 30 days prior to diagnosis, it ensures that as many as possible of those who actually had a colorectal cancer diagnosis based on an EP were included. As over 80% of those with an EP were admitted due to a condition related to a malignancy, the vast majority had an emergency onset diagnosis. Thus, the magnitude of a potential overestimation may be small. Secondly, detailed SACT information where pharmaceutical prescriptions were collected outside the hospital setting were not included in this study. Data linkage with the Prescription Registry would be required to add additional information. This limitation may bias our results as the number of SACT in this study may be an underestimation. This study also has several strengths. First, the study utilised comprehensive treatment information regarding all three treatment modalities: surgery, radiotherapy, and SACT. The study was also able to use individual-level information about income. And finally, the study used a population-based design and national, comprehensive, high quality data to generate results that are widely representative.

## Conclusion

This study showed that patients who were older, had advanced disease or increased comorbidities were more likely to have an emergency-onset diagnosis and less likely to receive tumour-related treatment. In general, income did not affect the waiting time or access to treatment among colorectal cancer patients. Therefore, public health awareness campaigns for colorectal cancer are important to ensure earlier hospital attendance, diagnosis, and best treatment in the Norwegian population.

## Supplementary Information

**Additional file 1.**

**Additional file 2.**

**Additional file 3.**

**Additional file 4.**

**Additional file 5.**

**Additional file 6.**

**Additional file 7.**

## Data Availability

The data that support the findings of this study are available upon request from the Cancer Registry of Norway, The Norwegian Patient Registry and Statistics Norway.

## Abbreviations

CCI: Charlson comorbidity index
CI: confidence interval
CPP: cancer patient pathways
CRN: Cancer Registry of Norway
EP: emergency presentation
NPR: Norwegian Patient Register
OR: Odds Ratio
SACT: systemic anticancer treatment
SES: socioeconomic status

## References

[1] World Health Organization. Global Health Observatory: World Health Organization; 2018 [Available from: https://gco.iarc.fr/today/data/factsheets/cancers/10_8_9-Colorectum-fact-sheet.pdf. Accessed: 01.11.2020.

[2] Cancer Registry of Norway. Cancer in Norway 2019 - Cancer incidence, mortality, survival and prevalence in Norway. Oslo, Cancer Registry of Norway; 2020.

[3] Arnold M, Rutherford MJ, Bardot A, Ferlay J, Andersson TM, Myklebust T (2019). Progress in cancer survival, mortality, and incidence in seven high-income countries 1995-2014 (ICBP SURVMARK-2): a population-based study. Lancet Oncol.

[4] Engholm G, Ferlay J, Christensen N, Bray F, Gjerstorff ML, Klint A (2010). NORDCAN-a Nordic tool for cancer information, planning, quality control and research. Acta oncologica (Stockholm, Sweden).

[5] Skyrud KD, Bray F, Eriksen MT, Nilssen Y, Møller B (2016). Regional variations in cancer survival: impact of tumour stage, socioeconomic status, comorbidity and type of treatment in Norway. International Journal of Cancer Journal international du cancer.

[6] Benitez Majano S, Di Girolamo C, Rachet B, Maringe C, Guren MG, Glimelius B (2019). Surgical treatment and survival from colorectal cancer in Denmark, England, Norway, and Sweden: a population-based study. Lancet Oncol..

[7] Zhou Y, Abel GA, Hamilton W, Pritchard-Jones K, Gross CP, Walter FM, Renzi C, Johnson S, McPhail S, Elliss-Brookes L, Lyratzopoulos G (2017). Diagnosis of cancer as an emergency: a critical review of current evidence. Nat Rev Clin Oncol.

[8] Helsedirektoratet. Pakkeforløp for tykk- og endetarmskreft [Available from: https://www.helsedirektoratet.no/pakkeforlop/tykk-og-endetarmskreft. Accessed: 25.02.2020.

[9] Nilssen Y, Brustugun OT, Eriksen MT, Haug ES, Naume B, Møller B (2020). Patient and tumour characteristics associated with inclusion in Cancer patient pathways in Norway in 2015-2016. BMC Cancer.

[10] Nilssen Y, Brustugun OT, Tandberg Eriksen M, Gulbrandsen J, Skaaheim Haug E, Naume B, Møller B (2019). Decreasing waiting time for treatment before and during implementation of cancer patient pathways in Norway. Cancer Epidemiol.

[11] McBride RB, Lebwohl B, Hershman DL, Neugut AI (2010). Impact of socioeconomic status on extent of lymph node dissection for colon cancer. Cancer Epidemiol Biomark Prev.

[12] Cavalli-Björkman N, Lambe M, Eaker S, Sandin F, Glimelius B (2011). Differences according to educational level in the management and survival of colorectal cancer in Sweden. European Journal of Cancer (Oxford, England : 1990).

[13] Hines R, Markossian T, Johnson A, Dong F, Bayakly R (2014). Geographic residency status and census tract socioeconomic status as determinants of colorectal cancer outcomes. Am J Public Health.

[14] Hsieh MC, Chiu YW, Velasco C, Wu XC, O'Flarity MB, Chen VW (2013). Impact of race/ethnicity and socioeconomic status on adjuvant chemotherapy use among elderly patients with stage III colon cancer. J Registry Manag.

[15] Larsen IK, Smastuen M, Johannesen TB, Langmark F, Parkin DM, Bray F (2009). Data quality at the Cancer Registry of Norway: an overview of comparability, completeness, validity and timeliness. European Journal of Cancer (Oxford, England : 1990).

[16] Kastpersen S, Kalseth B (2010). Omfang og Utvikling av det Selv-betalende Markedet for private Spesialisthelsetjenester i Norge [scope and development of the private spending on healthcare for private healthcare in Norway].

[17] Larsen IK, Myklebust TA, Johannesen TB, Moller B, Hofvind S (2018). Stage-specific incidence and survival of breast cancer in Norway: the implications of changes in coding and classification practice. Breast..

[18] OECD. What are equivalance scales [Available from: http://www.oecd.org/els/soc/OECD-Note-EquivalenceScales.pdf. Accessed: 06.02.2020.

[19] Charlson ME, Pompei P, Ales KL, MacKenzie CR (1987). A new method of classifying prognostic comorbidity in longitudinal studies: development and validation. J Chronic Dis.

[20] Nilssen Y, Strand TE, Wiik R, Bakken IJ, Yu XQ, O'Connell DL, Møller B (2014). Utilizing national patient-register data to control for comorbidity in prognostic studies. Clin Epidemiol.

[21] Direktoratet for e-helse. Prosedyrekodeverkene (Kodeverk for medisinske, kirurgiske og radiologiske prosedyrer, NCMP, NCSP og NCRP) [Available from: https://ehelse.no/kodeverk/prosedyrekodeverkene-kodeverk-for-medisinske-kirurgiske-og-radiologiske-prosedyrer-ncmp-ncsp-og-ncrp. Accessed: 01.12.2020.2020.

[22] Hosmer DW, Lemeshow S (2005). Applied logistic regression.

[23] Schmoll HJ, Van Cutsem E, Stein A, Valentini V, Glimelius B, Haustermans K (2012). ESMO consensus guidelines for management of patients with colon and rectal cancer. A personalized approach to clinical decision making. Ann Oncol.

[24] StataCorp (2019). Stata statistical software: release 16.

[25] Nesbakken A, Gaard M (2007). Surgical treatment of colon cancer. Tidsskr Nor Laegeforen.

[26] Gunnarsson H, Ekholm A, Olsson LI (2013). Emergency presentation and socioeconomic status in colon cancer. Eur J Surg Oncol.

[27] Papamichael D, Audisio RA, Glimelius B, de Gramont A, Glynne-Jones R, Haller D, Köhne CH, Rostoft S, Lemmens V, Mitry E, Rutten H, Sargent D, Sastre J, Seymour M, Starling N, van Cutsem E, Aapro M (2015). Treatment of colorectal cancer in older patients: International Society of Geriatric Oncology (SIOG) consensus recommendations 2013. Ann Oncol.

[28] Pilleron S, Charvat H, Araghi M, Arnold M, Fidler-Benaoudia MM, Bardot A, et al. Age disparities in stage-specific colon cancer survival across seven countries: an ICBP SURVMARK-2 population-based study. International Journal of Cancer Journal international du cancer. 2020. 10.1002/ijc.33326 33006395

[29] Petrelli F, Tomasello G, Borgonovo K, Ghidini M, Turati L, Dallera P, Passalacqua R, Sgroi G, Barni S (2017). Prognostic survival associated with left-sided vs right-sided Colon Cancer: a systematic review and meta-analysis. JAMA Oncol.

[30] Nasjonalt kvalitetsregister for tykk- og endetarmskreft. Årsrapport 2019 med resultater og forbedringstiltak fra Nasjonalt kvalitetsregister for tykk- og endetarmskreft. Kreftregisteret; 2020.

[31] Morris EJ, Finan PJ, Spencer K, Geh I, Crellin A, Quirke P (2016). Wide variation in the use of radiotherapy in the management of surgically treated rectal cancer across the English National Health Service. Clin Oncol (R Coll Radiol).

[32] Glynne-Jones R, Wyrwicz L, Tiret E, Brown G, Rödel C, Cervantes A (2017). Rectal cancer: ESMO Clinical Practice Guidelines for diagnosis, treatment and follow-up. Ann Oncol.

[33] Glimelius B, Myklebust T, Lundqvist K, Wibe A, Guren MG (2016). Two countries - two treatment strategies for rectal cancer. Radiotherapy and Oncology : Journal of the European Society for Therapeutic Radiology and Oncology.

[34] Åsli LM, Myklebust T, Kvaløy SO, Jetne V, Møller B, Levernes SG (2018). Factors influencing access to palliative radiotherapy: a Norwegian population-based study. Acta oncologica (Stockholm, Sweden).

