# Supplementary figures and images for: Factors associated with emergency-onset diagnosis, time to treatment and type of treatment in colorectal cancer patients in Norway
Source: BMC Cancer. 2021 Jun 30;21:757. doi: 10.1186/s12885-021-08415-1 (PMC8244161; doi:10.1186/s12885-021-08415-1)

# Proportion first and ever treated per age group

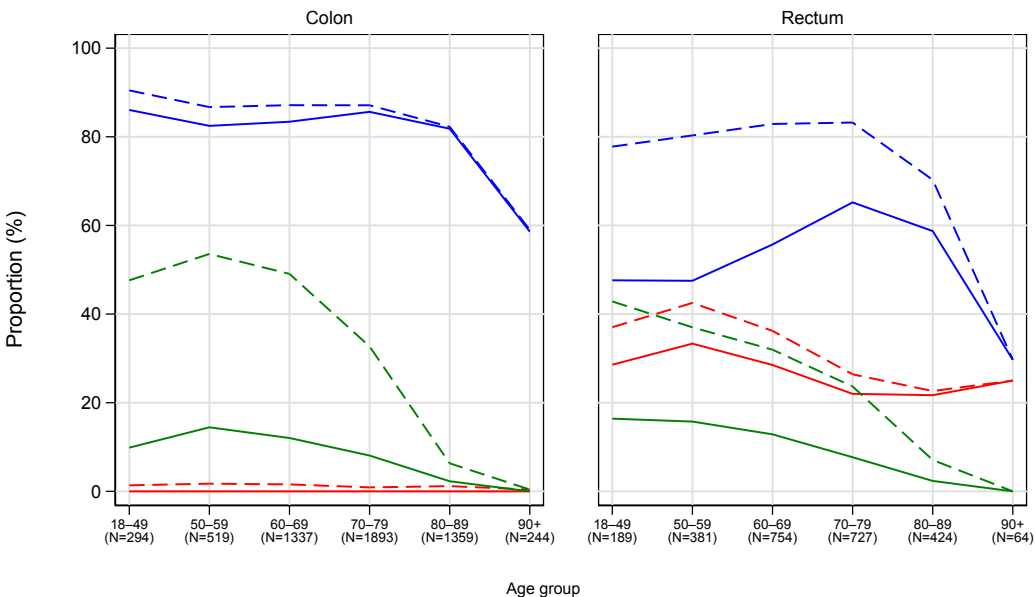

Supplement: Supplementary file 7 — Additional file 7. [file 12885_2021_8415_MOESM7_ESM.pdf]
